# Supplementary figures and images for: iPLA2-VIA is required for healthy aging of neurons, muscle, and the female germline in Drosophila melanogaster
Source: PLoS One. 2021 Sep 10;16(9):e0256738. doi: 10.1371/journal.pone.0256738 (PMC8432841; doi:10.1371/journal.pone.0256738)

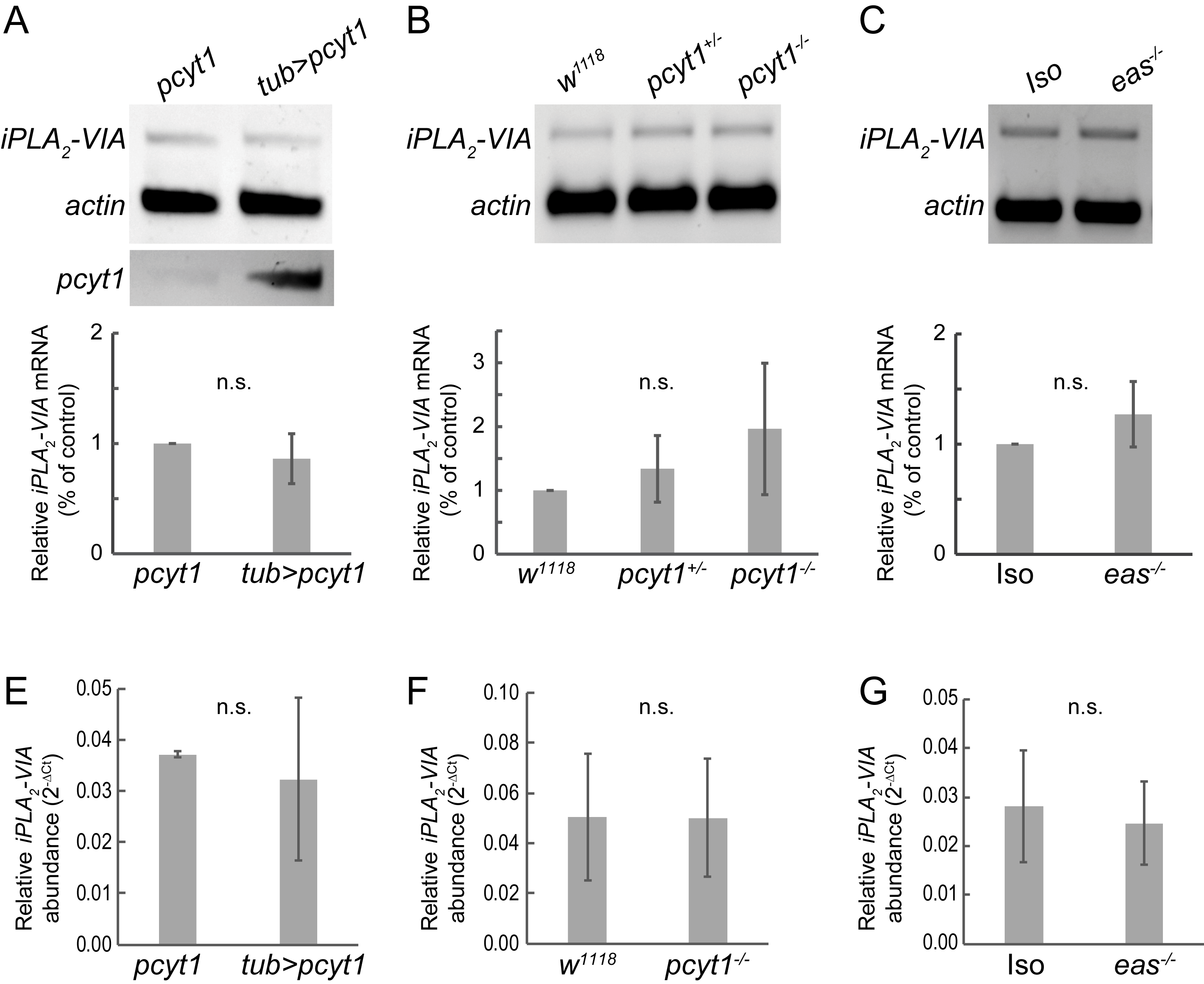

Supplement: S1 Fig — (A) Whole fly reverse transcription (RT) PCR shows no upregulation of iPLA2-VIA mRNA expression when Pcyt1 is overexpressed using tubulin-GAL4. Control siblings lack the tubulin-GAL4 driver. pcyt1 mRNA upregulation is shown in the bottom panel of the gel image. (B-C) Whole fly RT-PCR shows no downregulation of iPLA2-VIA expression in either the pcyt116919 mutant compared to w1118 (B) or in the easKO mutant compared to isogenic controls (C). Experiments were performed in triplicate. Quantifications were taken with Bio-Rad ImageLab 5.0, shown below representative gel images. Graphs show the ratio of iPLA2-VIA mRNA normalized to internal control actin mRNA in each mutant genotype compared to the control genotype, averaged across three biological replicates. Error bars are standard deviations. (E-G) Whole fly RT-qPCR confirms that iPLA2-VIA mRNA levels are not significantly different from controls when (E) Pcyt1 is overexpressed using tubulin-GAL4, (F) in pcyt116919 mutants, or (G) in easKO mutants. iPLA2-VIA mRNA abundance was normalized to rp49 mRNA abundance (2-ΔCt) and averaged across three biological replicates. Error bars are standard deviations. Statistical analysis by unpaired t-test (A, C, E-G) or single factor ANOVA (B), n.s. not significant. (TIF) [file pone.0256738.s001.tif]

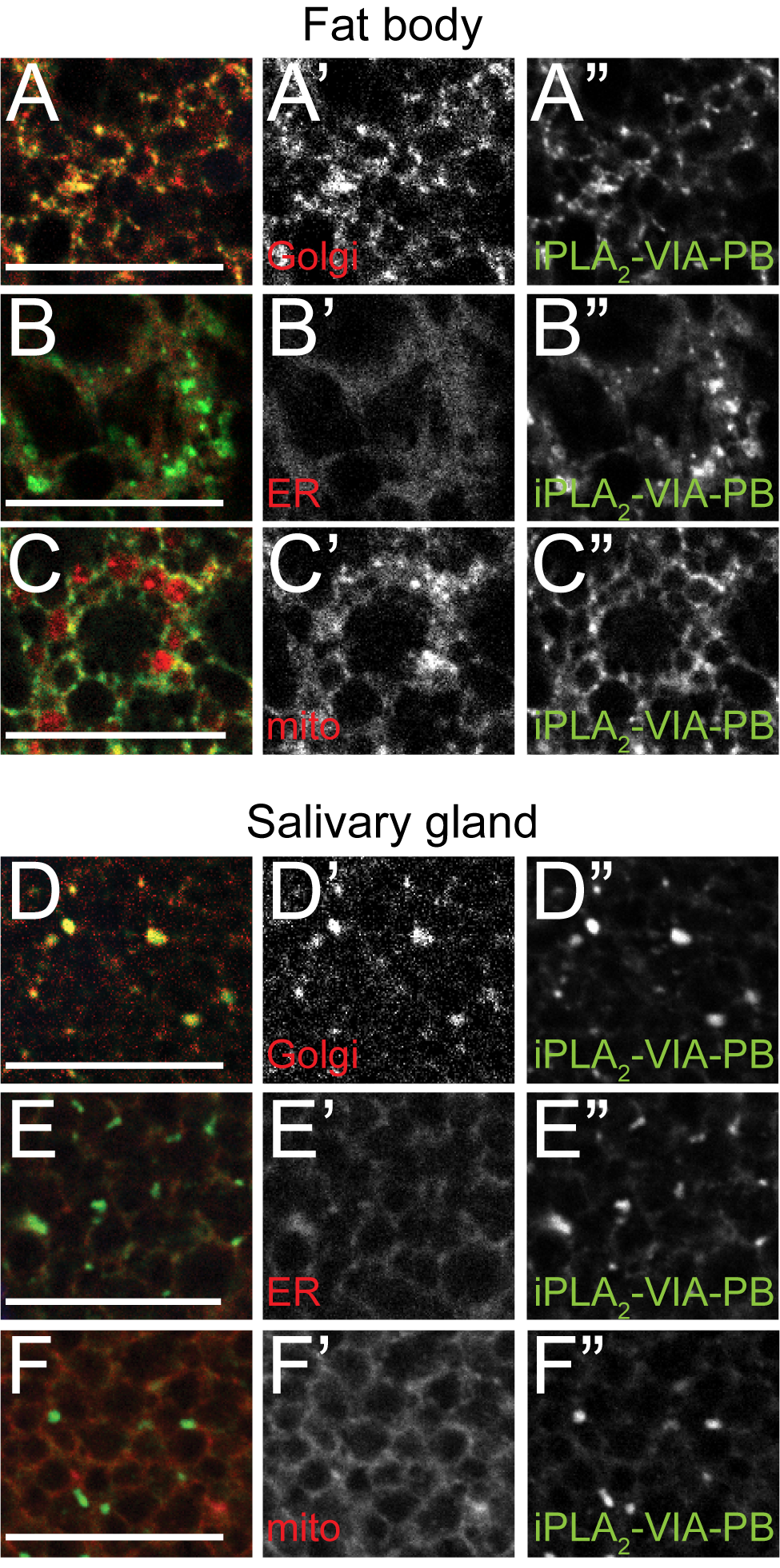

Supplement: S2 Fig — Five male and five female adult flies were collected for each of the following genotypes: tubulin-GAL4 > UAS-iPLA2-VIA-PB-WT; tubulin-GAL4 > UAS-iPLA2-VIA-PB-SA; tubulin-GAL4 alone. All three samples were processed in parallel, run on a denaturing SDS-PAGE gel, and blotted with both anti-HA to detect transgenic iPLA2-VIA-HA and with anti-beta-tubulin as a loading control. Representative blot shown in (A), quantification of three biological replicates shown in (B). iPLA2-VIA protein levels were normalized to beta-tubulin levels in each lane. Biological replicates are indicated by the gray circles, the average normalized protein levels are represented by the black bars, and error bars are standard deviations. (TIF) [file pone.0256738.s002.tif]

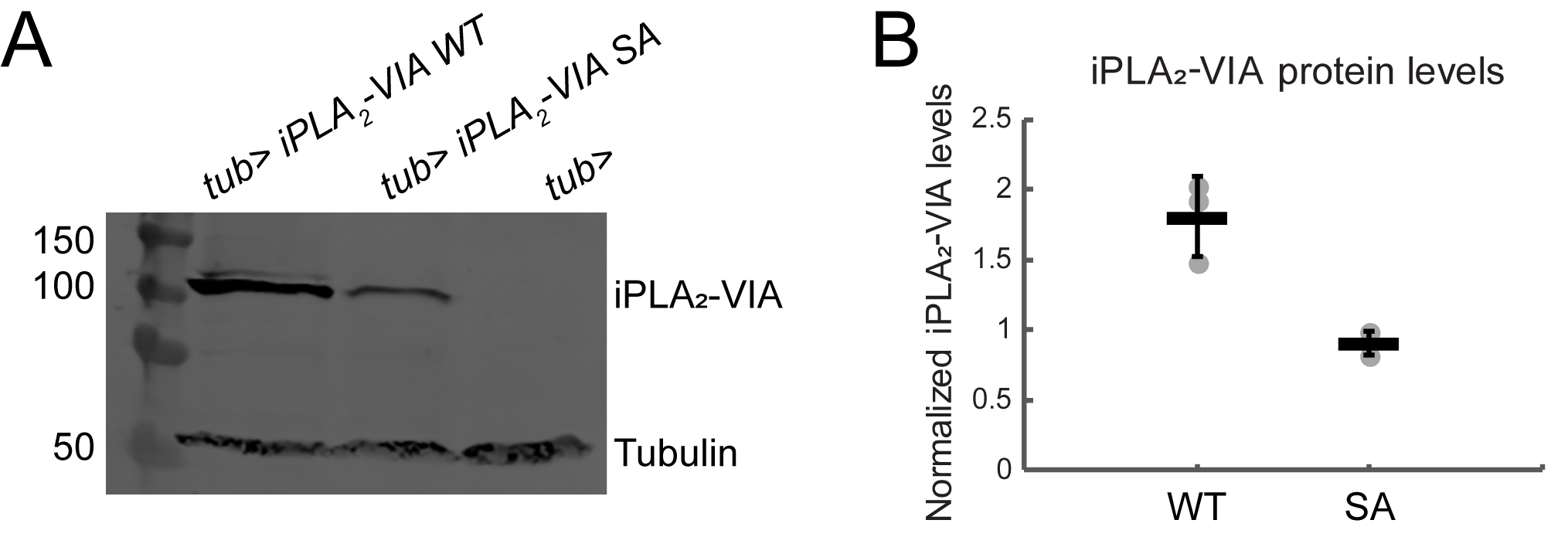

Supplement: S3 Fig — (A) Riboprobe antisense to wg transcript (arrows) was used to demonstrate the fidelity of imaginal disc in situ hybridizations. (B) Riboprobe antisense to cycB transcript was used to demonstrate fidelity of testis in situ hybridizations. Arrows indicate staining in primary spermatocytes, and arrowheads indicate meiotic spermatids. (C) The cycB riboprobe also revealed specific expression in the larval brain (arrows). Scale bars: 100 μm. (TIF) [file pone.0256738.s003.tif]

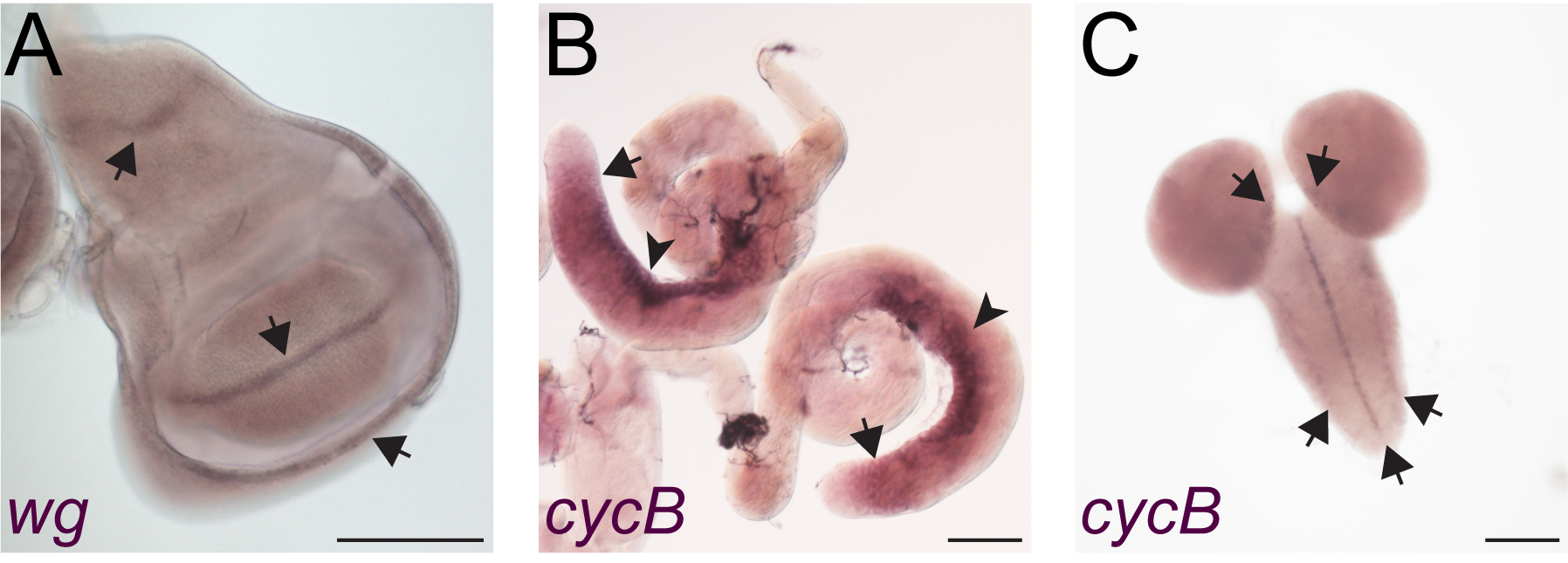

Supplement: S4 Fig — Young (<10 day old) homozygous iPLA2-VIAΔ23 females or isogenic control females were mated to control males and allowed to lay eggs on grape juice plates for 20 hours at 23°C. First instar larvae hatched from isolated eggs were counted (black bars). Second instar larvae molted from isolated first instars were counted (dark gray bars). Third instar larvae molted from isolated second instars were counted (medium gray bars). Pupae were counted from isolated third instars (light gray bars). The entire experiment was repeated three times. Bars represent the average percentage of individuals that progress to each stage in the three experiments. Error bars are standard deviations. No developmental lethality is observed in progeny from iPLA2-VIAΔ23 mutant mothers compared to control mothers. (TIF) [file pone.0256738.s004.tif]

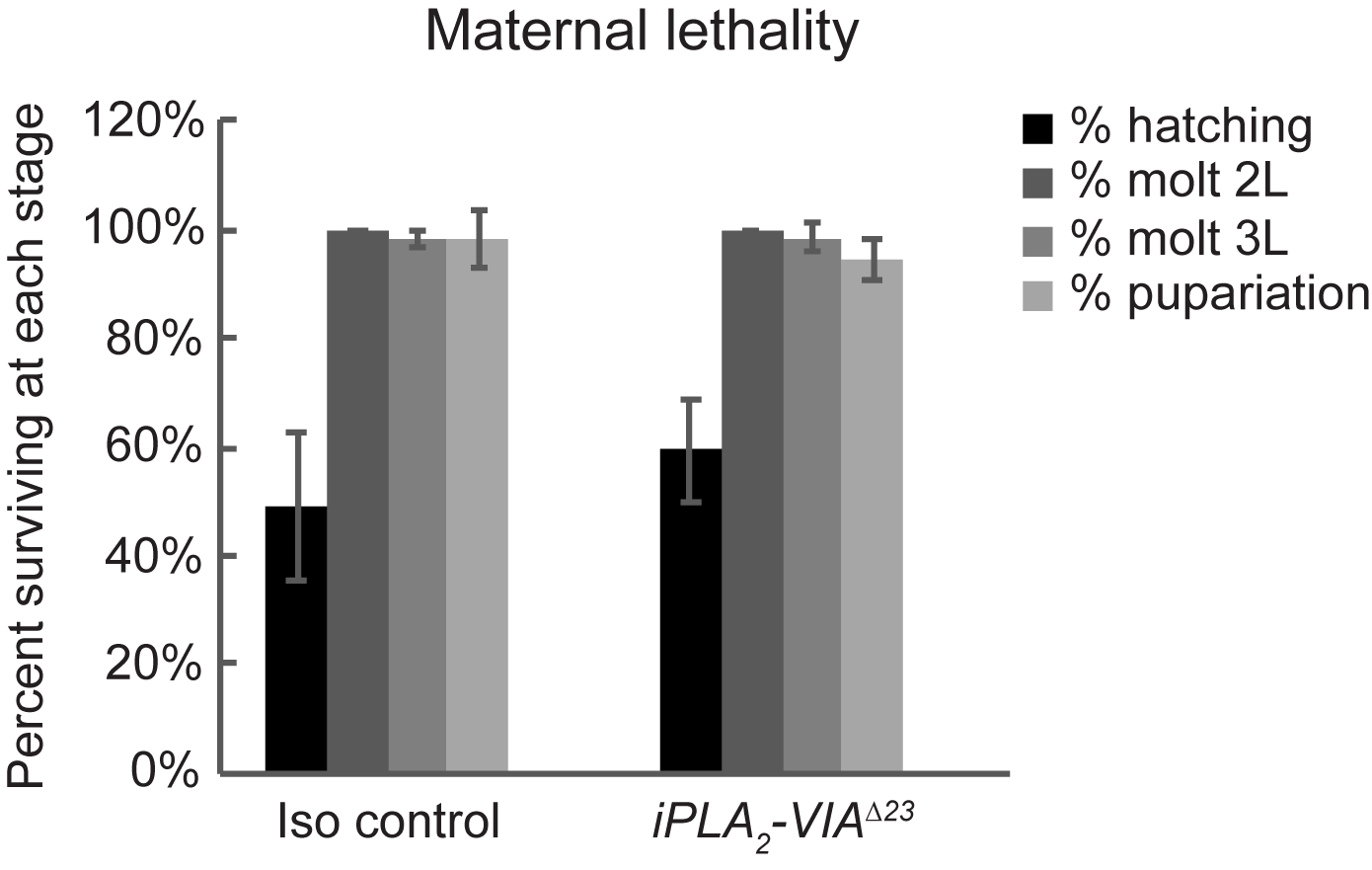

Supplement: S5 Fig — (A-B) Neither the parental stock carrying homozygous Psqh-mito-EYFP (A) nor iPLA2-VIAΔ23 heterozygotes (B, Psqh-mito-EYFP, iPLA2-VIAΔ23/revertantΔ11) show mitochondrial clumping even at 6 weeks of age (white, mito-YFP; magenta, DAPI). (C-D) Mitochondrial clumping also is observed with another marker, immunofluorescence to ATP-5A protein in iPLA2-VIAΔ23 mutants (D) but not in isogenic controls (C) at five weeks of age (white, anti-ATP-5A; magenta, DAPI). Magnified views of the boxed regions are shown in the insets. (E-F) Additionally, the ATP-5A signal is weaker in iPLA2-VIAΔ23 mutants (dark red bars) than in age-matched controls (dark gray bars) at four (E) and five (F) weeks of age, possibly indicating mitochondrial degradation in the mutant. Bars represent averages, error bars are standard deviations. Statistical comparison by unpaired t-test. Scale bars: 20 μm. (TIF) [file pone.0256738.s005.tif]

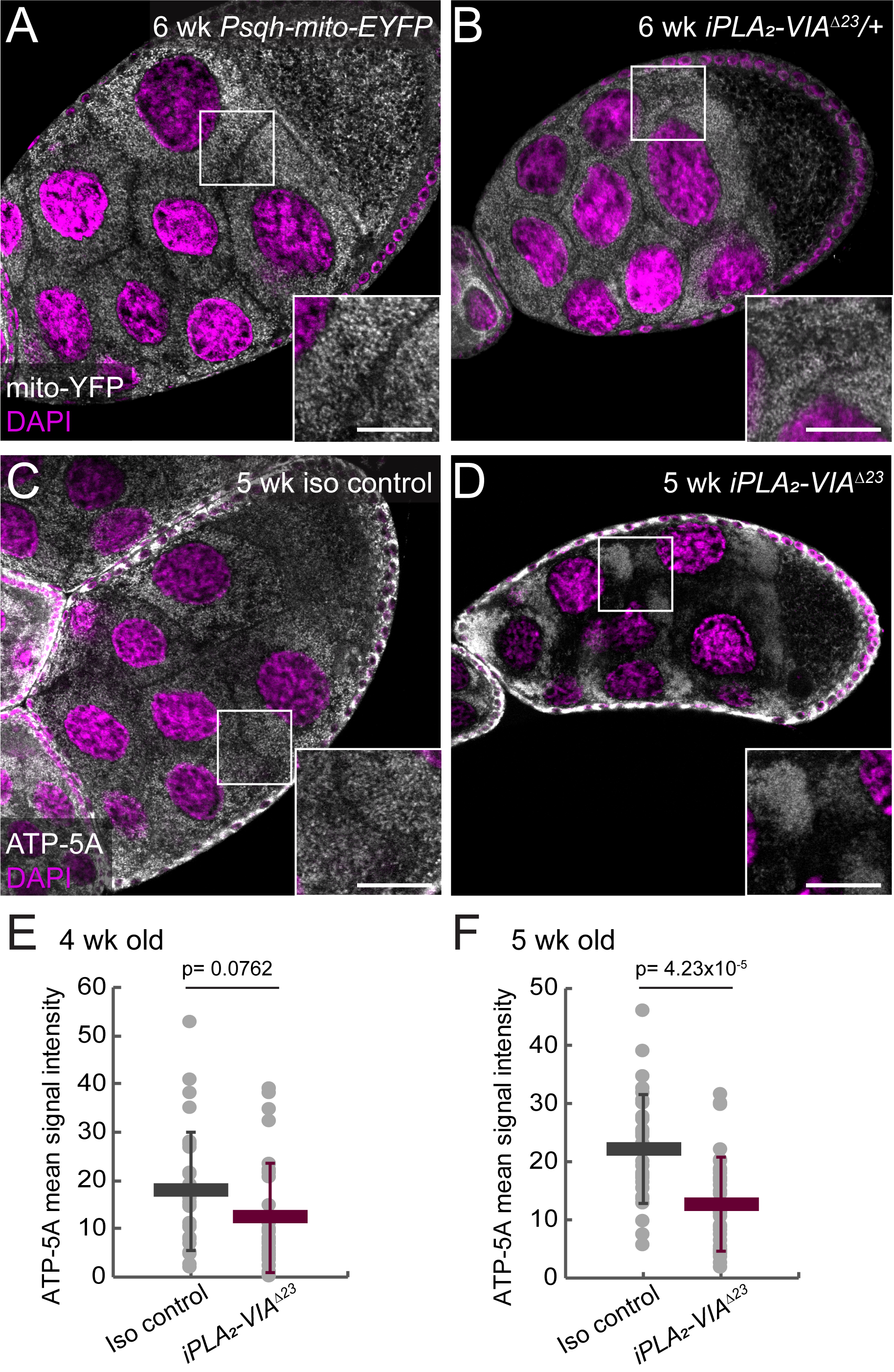

Supplement: S6 Fig — HA-tagged wild-type iPLA2-VIA-PB transgenic protein appears in puncta (green, A”-F”, expressed with tubulin-GAL4) that colocalize with a Golgi marker (red, anti-Golgin 84) in larval fat body (A) and salivary glands (D). Colocalization with an ER marker (red, B, E, anti-Calnexin 99A) and a mitochondrial marker (red, C, F, UAS-mCherry-mitoOMM) is weak or undetectable in these larval tissues. Scale bars: 20 μm. (TIF) [file pone.0256738.s006.tif]
