# Supplementary material for: iPLA2-VIA is required for healthy aging of neurons, muscle, and the female germline in Drosophila melanogaster
Source: PLoS One. 2021 Sep 10;16(9):e0256738. doi: 10.1371/journal.pone.0256738 (PMC8432841; doi:10.1371/journal.pone.0256738)

$iPLA_2$-VIA is required for healthy aging of neurons, muscle, and the female germline in *Drosophila melanogaster*

Surya Jyoti Banerjee†, Adina Schonbrun†, Sogol Eizadshenass, Shimshon Benji, Yaakov Tzvi Cantor, Liam Eliach, Matthew Lubin, Zev Narrowe, Jeremy Purow, Benjamin Shulman, Leib Wiener, and Josefa Steinhauer*

Raw gel and blot images

Figure 1. Agarose gel images captured with Bio-Rad Chemi-Doc MP Imaging System.

25 cycle RT-PCR

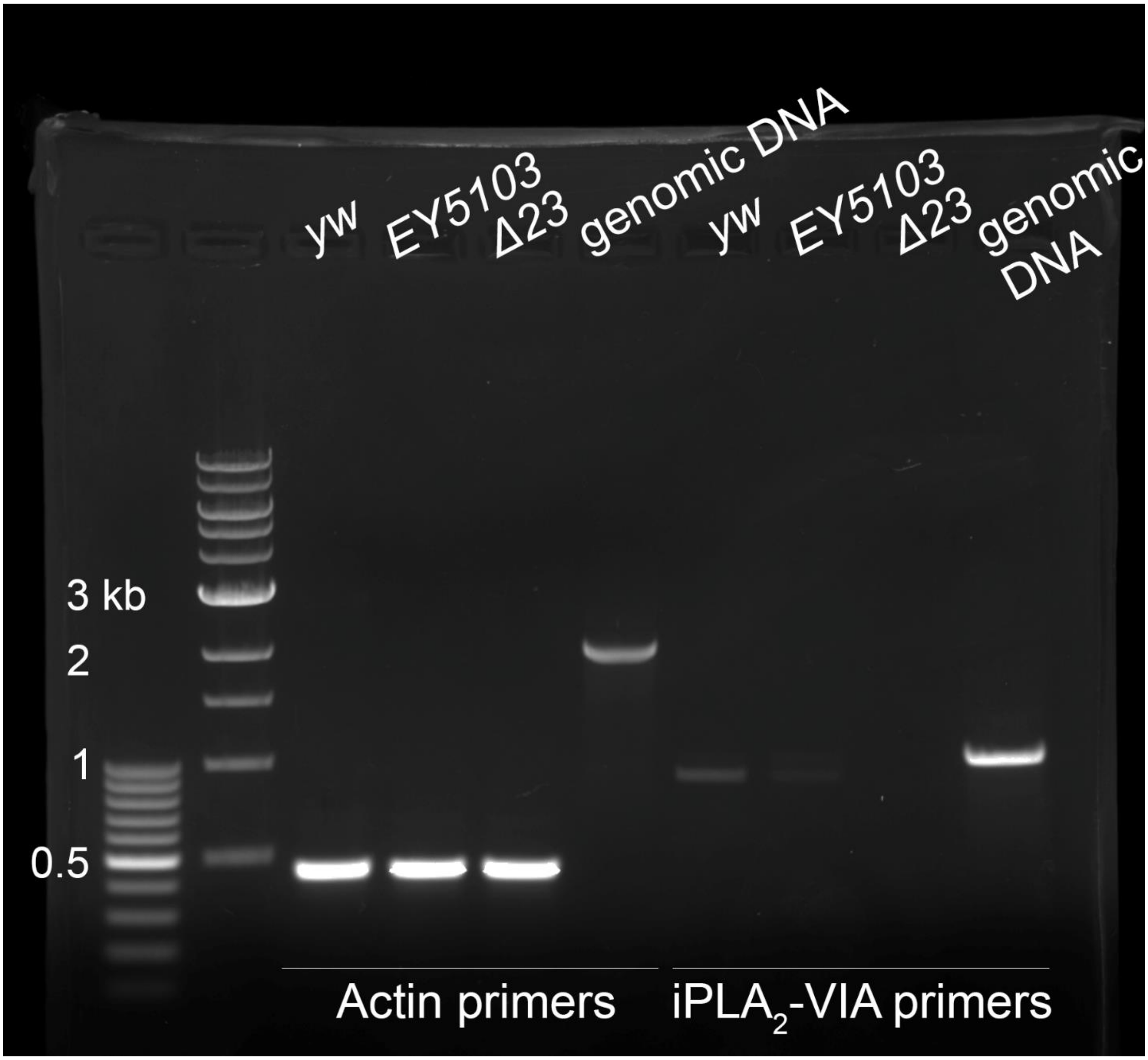

30 cycle RT-PCR

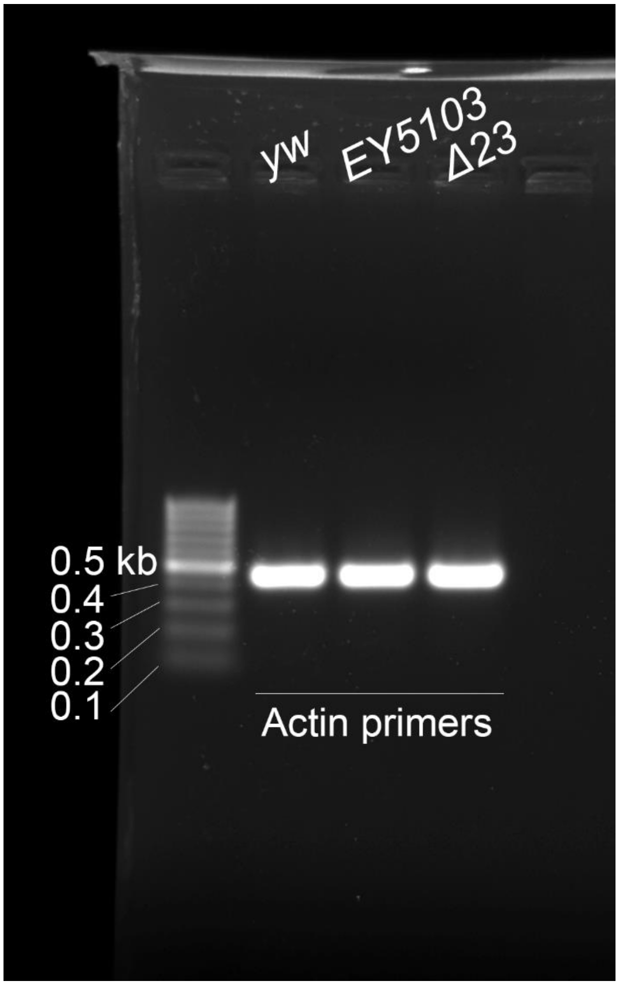

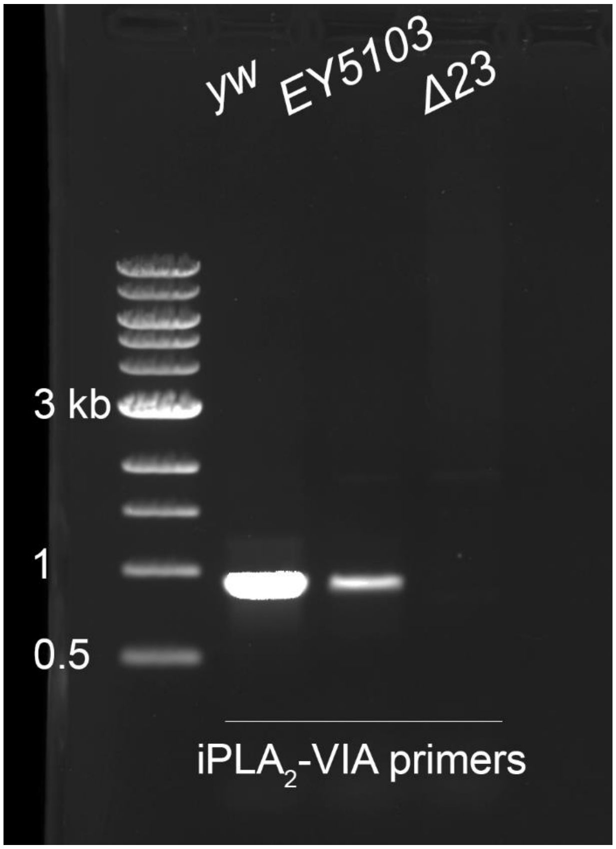

Figure S1. Agarose gel images captured with Bio-Rad Chemi-Doc MP Imaging System.

A.

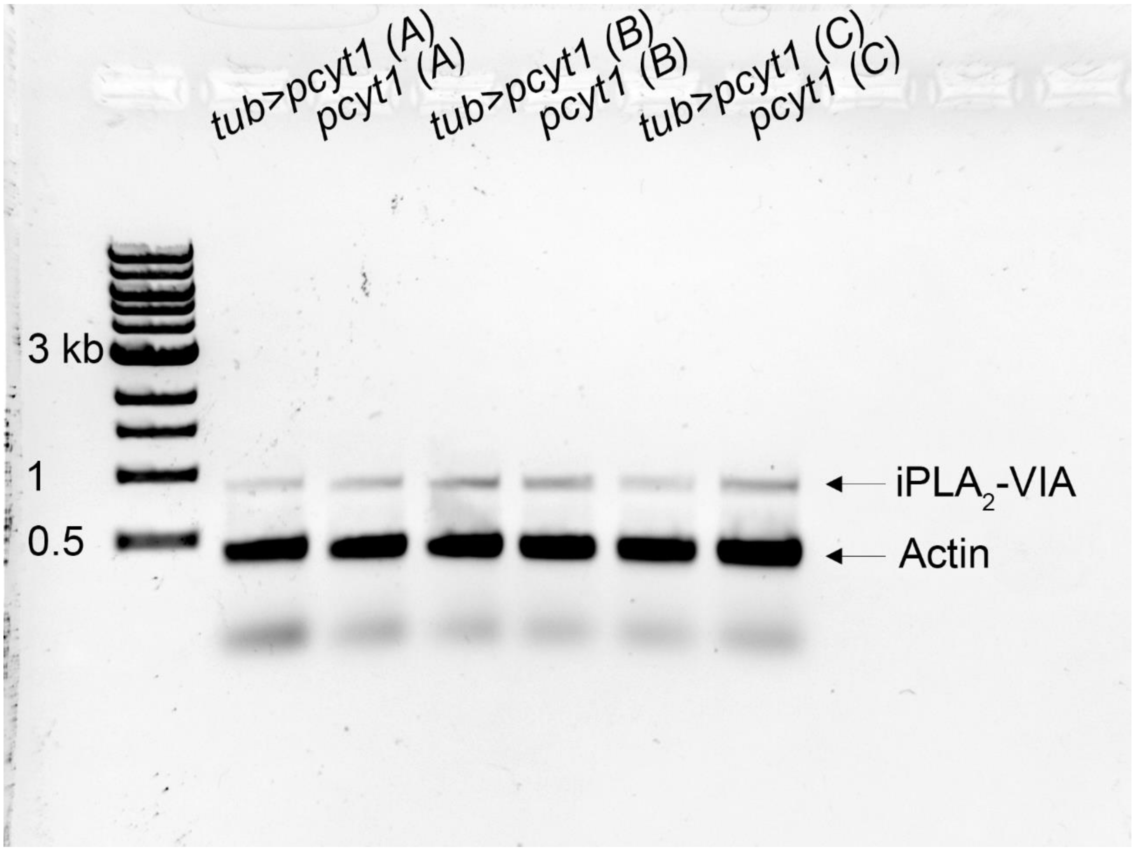

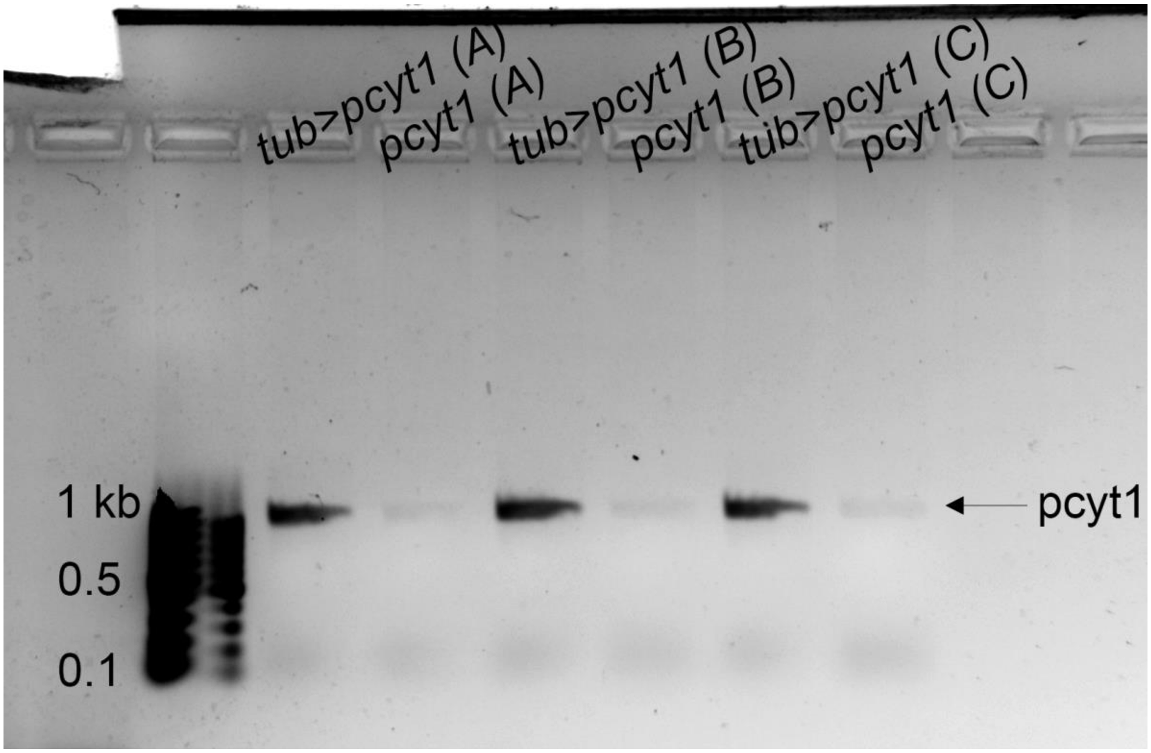
tub>pcyt1 (A)
pcyt1 (A)
tub>pcyt1 (B)
pcyt1 (B)
tub>pcyt1 (C)
pcyt1 (C)
1 kb
0.5
0.1
pcyt1

B.

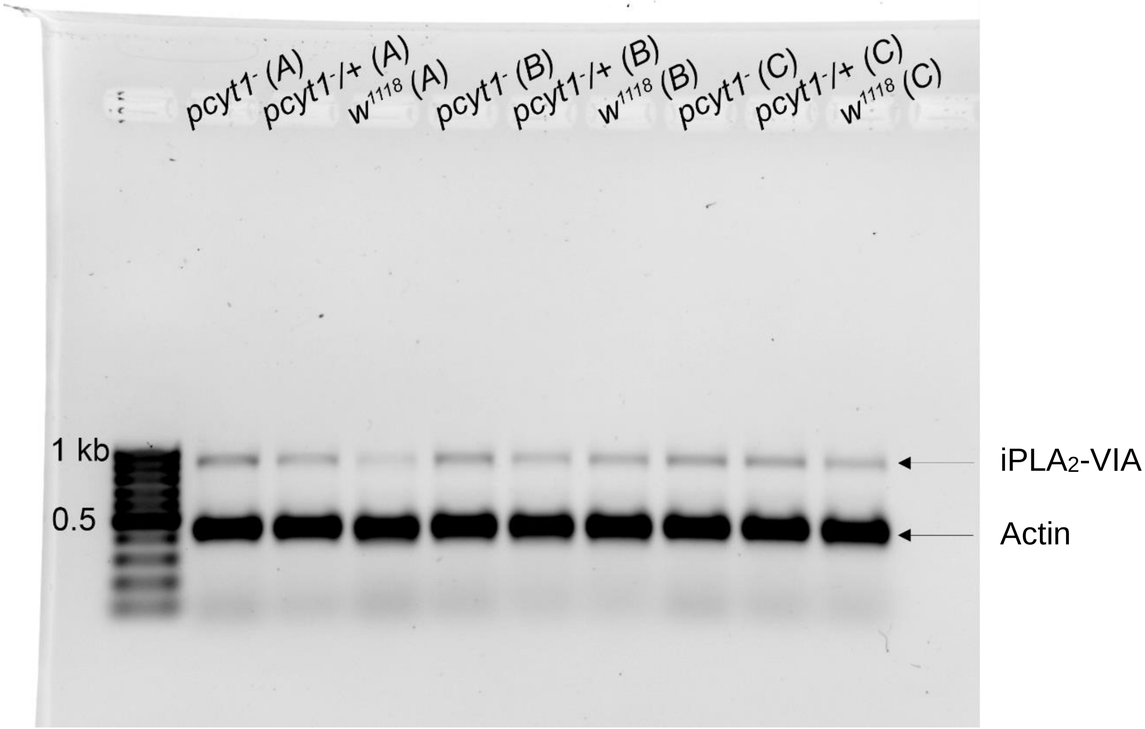
pcyt1- (A)
pcyt1-/+ (A)
w1118 (A)
pcyt1- (B)
pcyt1-/+ (B)
w1118 (B)
pcyt1- (C)
pcyt1-/+ (C)
w1118 (C)
1 kb
0.5
iPLA2-VIA
Actin

C.

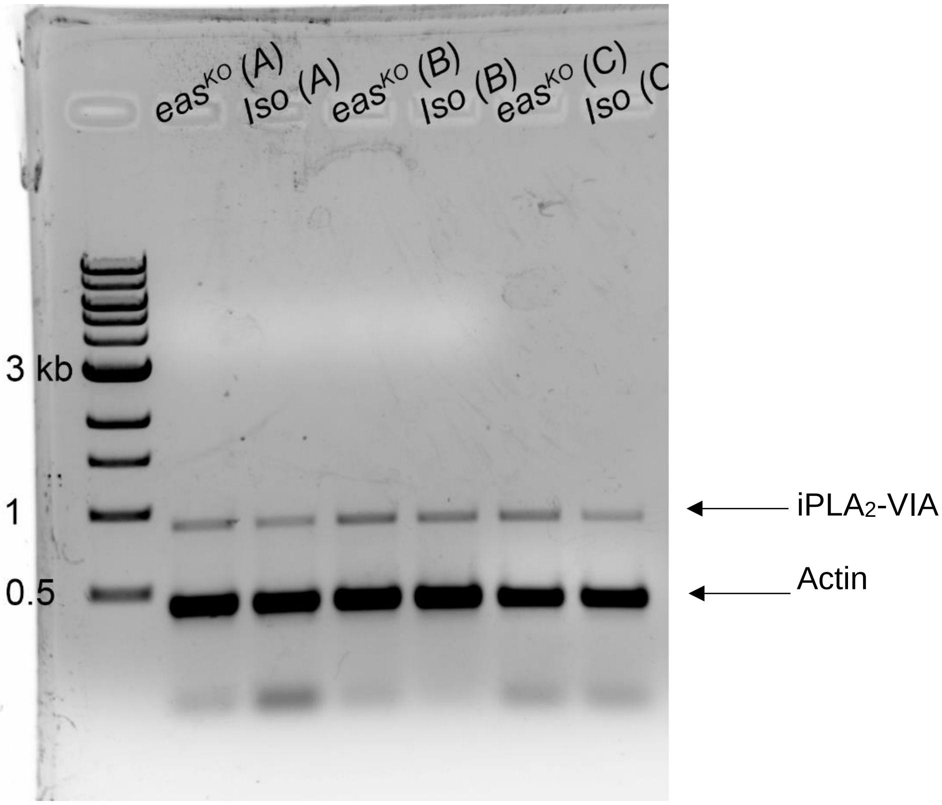

Figure S2. Western blot captured with Li-Cor Odyssey.

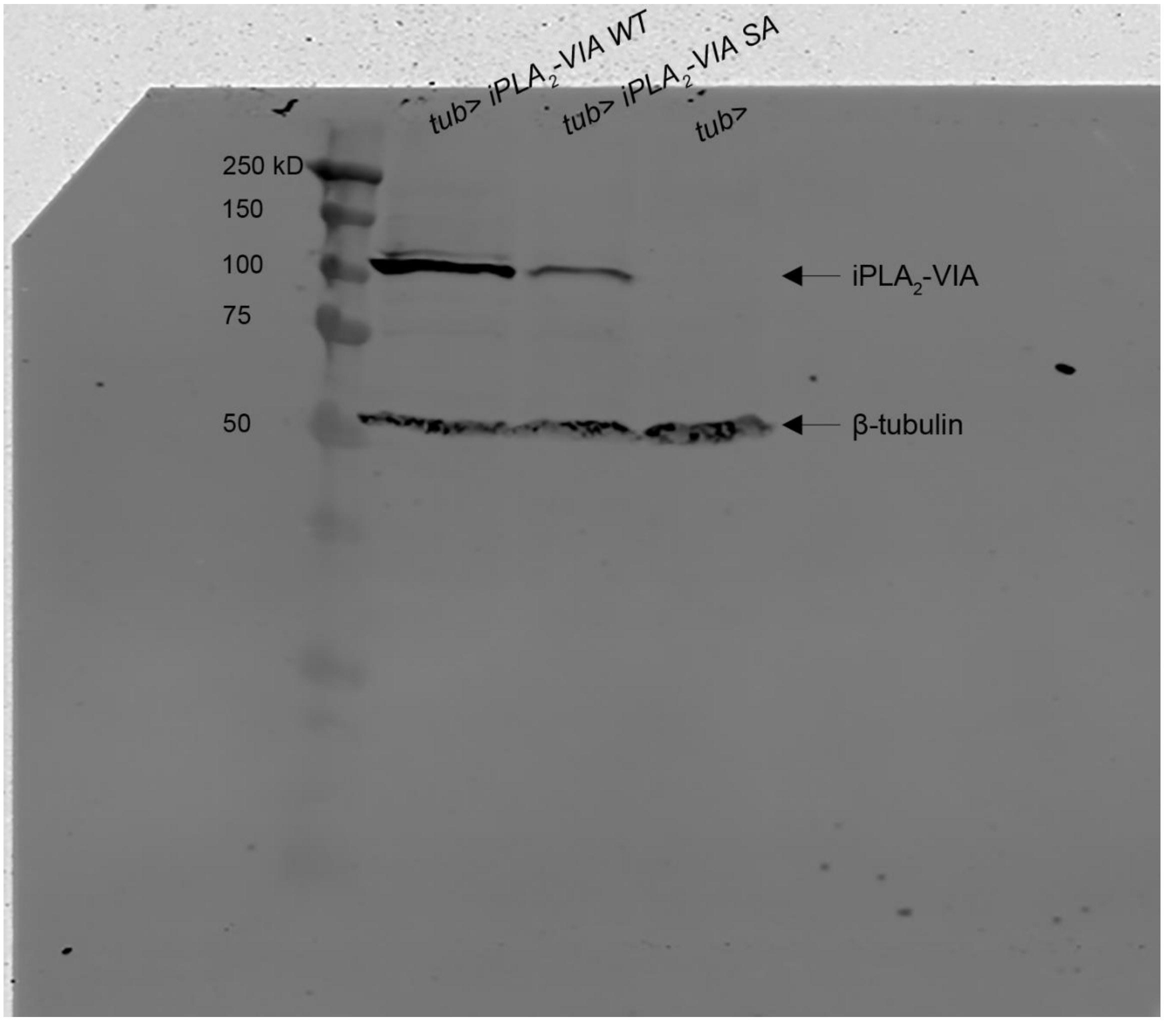

Supplement: S1 File — (PDF) [file pone.0256738.s007.pdf]
